# Supplementary figures and images for: From morphology to molecules: a combined source approach to untangle the taxonomy of Clessinia (Gastropoda, Odontostomidae), endemic land snails from the Dry Chaco ecoregion
Source: PeerJ. 2018 Dec 6;6:e5986. doi: 10.7717/peerj.5986 (PMC6286805; doi:10.7717/peerj.5986)

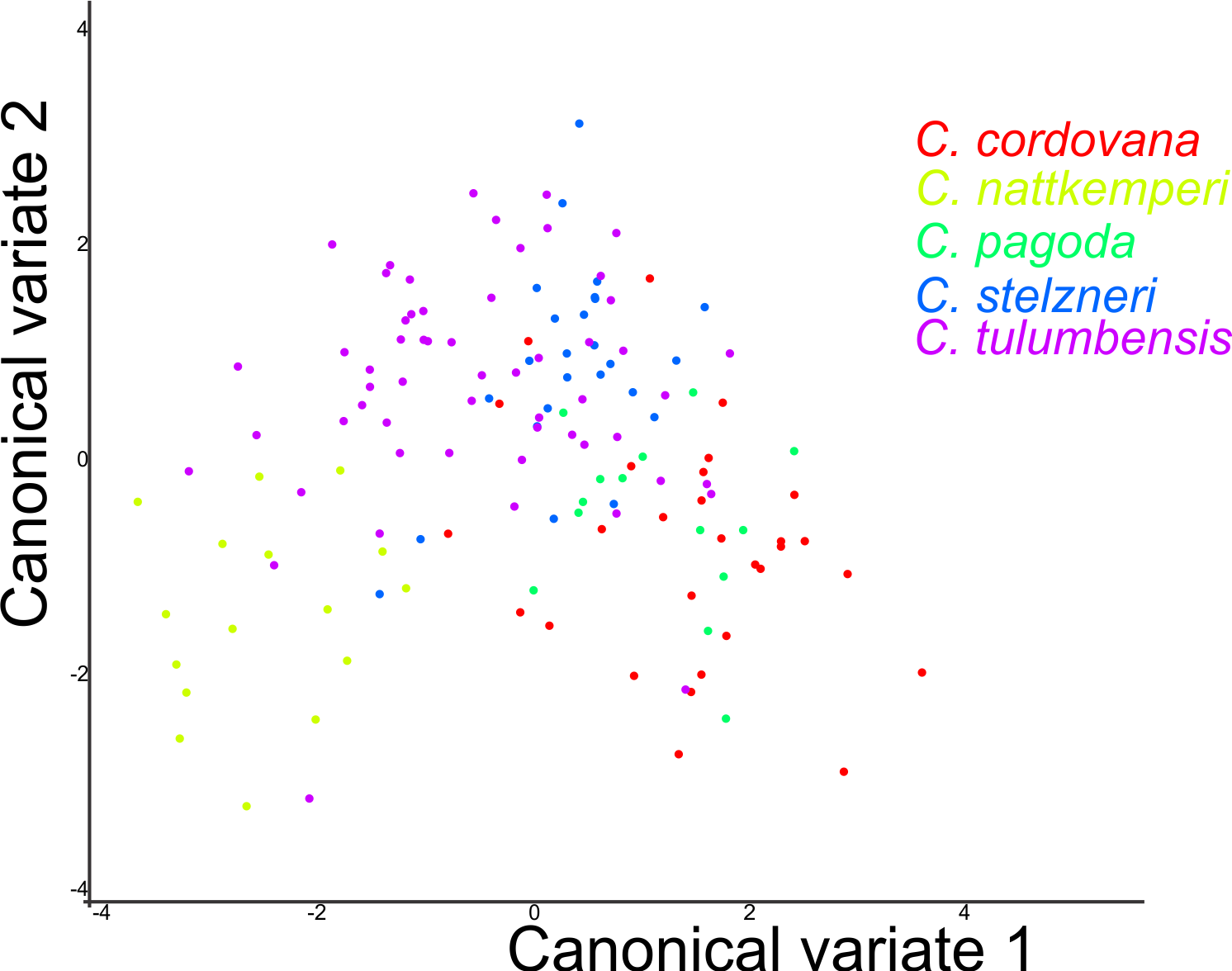

Supplement: Supplemental Information 1 — Coordinates of the landmarks in two dimensions, digitized with Tpsdig 2, selected in lateral view of the shell of the species of Clessinia (C. cordovana, C. stelzneri, C. tulumbensis, C. nattkemperi and C. pagoda). [file peerj-06-5986-s001.png]

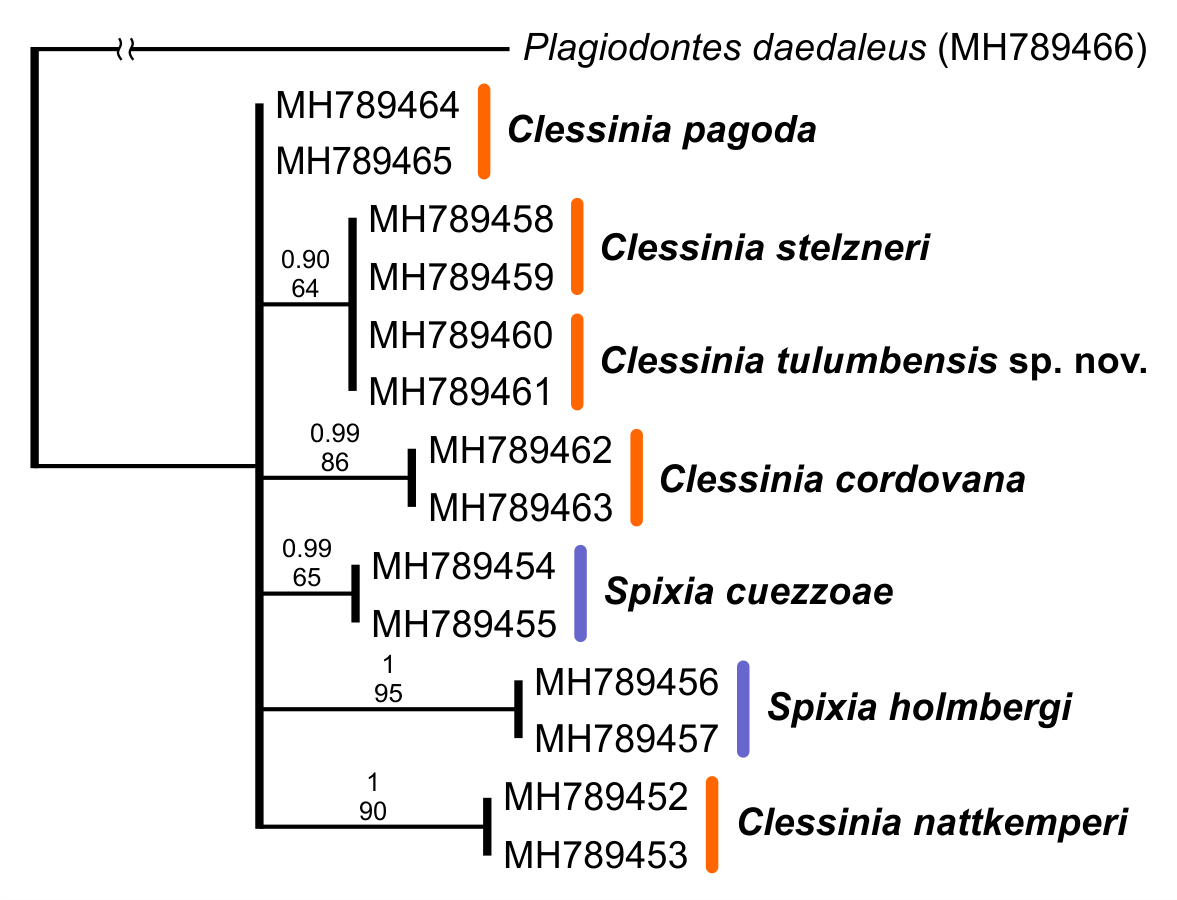

Supplement: Supplemental Information 2 — The posterior-probability values for BI and bootstrap values for the ML tree are shown above the branches. Numbers within groups are GenBank accession numbers. [file peerj-06-5986-s002.png]
