# Supplementary material for: From morphology to molecules: a combined source approach to untangle the taxonomy of Clessinia (Gastropoda, Odontostomidae), endemic land snails from the Dry Chaco ecoregion
Source: PeerJ. 2018 Dec 6;6:e5986. doi: 10.7717/peerj.5986 (PMC6286805; doi:10.7717/peerj.5986)
Supplement: Supplemental Information 3 — Material from different malacological collections studied. Abbreviations: IBN, Instituto de Biodiversidad Neotropical, Tucumán, Argentina; IFML-Moll, Instituto-Fundación Miguel Lillo, Tucumán, Argentina; MACN-In, Museo Argentino de Ciencias Naturales, Buenos Aires, Argentina. [file peerj-06-5986-s003.docx]

| Species | Collection lote | Province | Department | Locality | Height | Collector and Date of collection |
| --- | --- | --- | --- | --- | --- | --- |
| *Clessinia cordovana* (Pfeiffer) | IFML-Moll 13521 | Córdoba | Pocho | on the road from Las Palmas to Chancani | 1,050 m | WW. Weyrauch |
|  | IFML-Moll 15415 | Córdoba | Pocho | Sierra de Pocho | 1,050 m | W.W. Weyrauch, 02/12/1969 |
|  | IFML-Moll 15413-15414 | Córdoba | Pocho | on the road from Las Palmas to Chancani | 1,050 m | WW. Weyrauch |
|  | IFML-Moll 15240 | Córdoba | Pocho | on the road from Las Palmas to El Cadillo (31º22’01”S, 65º24’7”W) | 869 m | MG. Cuezzo & E. Salas Oroño, 18/03/2006 |
|  | IBN 375 | Córdoba | Pocho | 13 km from El Cadillo (31°22’60”S, 65°23’30”W) | 1,440 m | MG. Cuezzo & E. Salas Oroño, 18/03/2006 |
|  | IBN 574 | Córdoba | Cruz del Eje | San Marcos Sierra, margin of the river through the town (30°46’48”S, 64°37’45.8”W) | 641 m | MG. Cuezzo & E. Salas Oroño, 26/11/2008 |
|  | IBN 563 | Córdoba | Cruz del Eje | San Marcos Sierra, Cerro de La Cruz (30º47’16.2”S, 64º37’56.6”W) | 683 m | MG. Cuezzo & E. Salas Oroño, 26/11/2008 |
|  | IBN 717 | Córdoba | Cruz del Eje | San Marcos Sierra (30º47’31.6”S, 64º37’55.7”W) | 742 m | MG. Cuezzo & E. Salas Oroño, 26/11/2008 |
|  | MACN-In 1593 | Córdoba | Cruz del Eje | San Marcos Sierra |  |  |
|  | IBN 891 | Córdoba | Cruz del Eje | San Marcos Sierra (30º47’50”S, 64º37’46.47”W) | 832 m | MG. Cuezzo, 20/10/2017 |
|  | IBN 886 | Córdoba | Cruz del Eje | San Marcos Sierra, Cerro de la Cruz (30º47’12.6”S, 64º30’2.5”W) | 680 m | MG. Cuezzo, 20/10/2017 |
|  | IFML-Moll 15768A | Córdoba | Tulumba | route 16 (30°24’00.8”S, 64°02’32”W) | 633 m | MG. Cuezzo & E. Salas Oroño, 25/11/2008 |
|  | MACN-In 421 | Catamarca | Ambato | Puerta de Ambato |  |  |
|  | MACN-In 9127 | Córdoba |  |  |  |  |
|  | MACN-In 19624 | Córdoba |  |  |  |  |
| *Clessinia stelzneri* (Doering) | IFML-Moll 10739 | Córdoba | Tulumba | Cerro San Vicente, 6 km east from Dean Funes | 850 m | WW. Weyrauch, 25/12/1965 |
|  | MACN-In 9226 | Córdoba | Tulumba | route 16, between Dean Funes and Tulumba |  |  |
|  | IBN 560 | Córdoba | Tulumba | route 16, between Dean Funes and Tulumba (30º26’19.6”S, 64º17’08.8”W) | 835 m | MG. Cuezzo & E. Salas Oroño, 24/11/2008 |
|  | IBN 323 | Córdoba | Tulumba | 20 km to Tulumba | - | MG. Cuezzo, 14/01/2005 |
|  | IBN 882 | Córdoba | Tulumba | Cerro San Vicente (30º25’44.7”S, 64º14’49.57”W) | 933 m | MG. Cuezzo, 21/10/2017 |
| *Clessinia tulumbensis* sp.nov. | IFML-Moll 14745A | Córdoba | Tulumba | 20 km from Dean Funes to Tulumba |  | E. Salas Oroño, 14/01/2005 |
|  | IBN 377 IFML-Moll 15245 | Córdoba | Tulumba | Route 16 from San José de La Dormida to Tulumba (30º24’0.4”S, 64º2’4.5”W) | 616 m | E. Salas Oroño, 16/03/2006 |
|  | IFML-Moll 16895 | Córdoba | Tulumba | Between Tulumba and San Jose de la Dormida, route 16 (30º22’24.2”S, 63º58’51.7”W) | 523 m | MG Cuezzo, 25/11/2008 |
|  | IFML-Moll 13516 | Córdoba | Tulumba | Virgen de Fátima, 34 km north from Villa General Mitre (Villa del Totoral), on the road to Villa María | 400 m | WW. Weyrauch, 10/02/1970 |
|  | IBN 575 | Córdoba | Tulumba | Route 16 (30º24’0.8”S, 64º02’32”W) | 633 m | MG. Cuezzo & E. Salas Oroño, 25/11/2008 |
|  | IBN 558 | Córdoba | Tulumba | Route 16 (30º24’3.4”S, 64º02’2.5”W) | 628 m | MG. Cuezzo & E. Salas Oroño, 24/11/2008 |
|  | IBN 373 | Córdoba | Tulumba | San José de La Dormida (30º22’36”S, 63º58’82”W) | 433 m | MG. Cuezzo & E. Salas Oroño, 16/03/2006 |
|  | IBN 571 | Córdoba | Tulumba | Route 16 between Tulumba village and San Jose de La Dormida (30º24’0.8”S, 64º02’32”W) | 633 m | MG. Cuezzo & E. Salas Oroño, 25/11/2008 |
|  | IBN 94S | Córdoba | Tulumba | On the road to Cerro Colorado close to Charqui Cañada (30º09’27.75”S, 63º55’51.63”W) | 588 m | MG. Cuezzo & E. Domínguez, 20/01/2017 |
| *Clessinia pagoda* Hylton Scott | IFML-Moll 15178 | Córdoba | Cruz del Eje | San Marcos Sierra |  | Cichero-Biraben & M.I. Hylton Scott, 3/4/1967 |
|  | IFML-Moll 15237 | Córdoba | Cruz del Eje | San Marcos Sierra, Cerro de la Cruz (30º47’22”S, 64º37’96”W) | 677 m | MG. Cuezzo & E. Salas Oroño, 17/03/2006 |
|  | IBN 564 | Córdoba | Cruz del Eje | San Marcos Sierra, Cerro de la Cruz (30º47’16.2”S, 64º37’56.6”W) | 690 m | MG. Cuezzo & E. Salas Oroño, 26/11/2008 |
|  | IBN 567 | Córdoba | Cruz del Eje | San Marcos Sierra, Cerro de la Cruz (30º47’37.7”S, 64º31’43.4”W) | 780 m | MG. Cuezzo & E. Salas Oroño, 26/11/2008 |
|  | IBN 376 | Córdoba | Cruz del Eje | San Marcos Sierra, Cerro de La Cruz (30º47’22”S, 64º37’96”W) | 677 m | MG. Cuezzo, 17/03/2006 |
|  | IBN 890 | Córdoba | Cruz del Eje | San Marcos Sierra (64º37’ 46.47”S, 30º47’50”W) | 832 m | MG. Cuezzo, 21/10/2017 |
|  | IFML-Moll 15763 | Córdoba | Cruz del Eje | Estancia La Fronda, 13 km from San Marcos Sierra | 620 m | WW. Weyrauch, 25/XII/1967 |
|  | IFML-Moll 15178 | Córdoba | Cruz del Eje | San Marcos Sierra |  | Birabén-Cichero, 5/IV/1967 |
|  | IFML-Moll 15759A | Córdoba | Cruz del Eje | San Marcos Sierra, Cerro de la Cruz, in a mountain on the side of the road, under rocks (30º47’22”S, 64º37’96”W) | 677 m | MG. Cuezzo & E. Salas Oroño, 17/03/2006 |
|  | IBN 904 | Córdoba | Cruz del Eje | Quilpo (30º51’21.36”S, 64º40’31.99”W) | 565 m | MG. Cuezzo & E. Domínguez, 21/10/2017 |
| *Clessinia nattkemperi* (Parodiz) | IBN 664 | Catamarca | Esquiú | Pomancillo del Este (28º17’47”S, 65º43’17”W) | 675 m | MG. Cuezzo, 11/11/2007 |
|  | IBN 374 | Catamarca | Esquiú | Pomancillo (28º17’76”S, 65º43’33”W) | 685 m | MG. Cuezzo, 15/03/2006 |
|  | IFML-Moll 15186A, IBN 475 | Catamarca | Esquiú | Pomancillo del Este (28º17’47”S, 65º48’17”W) | 675 m | MG. Cuezzo & E. Salas Oroño |
|  | IFML-Moll 15185A | Catamarca | Esquiú | Pomancillo, Sierra de Graciana, 23 km from San Fernando de Catamarca | 685 m | MG. Cuezzo & E. Salas Oroño, 15/03/2006 |
|  | IFML-Moll 11058 | Catamarca | Esquiú | Pomancillo, 25 km north of Catamarca | 650 m | WW. Weyrauch, 30/11/1967 |
|  | IFML-Moll 15193A | Catamarca | Esquiú | Pomancillo del Este, margin of route 1 (28º17’47”S, 65º48’17”W) | 675 m | MG. Cuezzo, 16/11/2007 |
|  | IBN 878 | Catamarca | Esquiú | Pomancillo del Oeste (28º18’43.9”S, 65º43’00.9”W) | 652 m | MG. Cuezzo, C. Tomassi & S. Albanesi, 22/9/2017 |
